# The mysterious link between migraine aura and migraine headache

**DOI:** 10.1371/journal.pbio.3003168

**Published:** 2025-06-11

**Authors:** Anders Hougaard, Cenk Ayata, K.C. Brennan, Arn M.J.M. van den Maagdenberg, Messoud Ashina

**Affiliations:** 1 Department of Neurology, Copenhagen University Hospital – Herlev and Gentofte, Herlev, Denmark; 2 Department of Clinical Medicine, Faculty of Health and Medical Sciences, University of Copenhagen, Copenhagen, Denmark; 3 Stroke Service, Department of Neurology, and Neurovascular Research Unit, Department of Radiology, Massachusetts General Hospital, Harvard Medical School, Charlestown, Massachusetts, United States of America; 4 Headache Physiology Laboratory, Department of Neurology, University of Utah, Salt Lake City, Utah, United States of America; 5 Department of Human Genetics, Leiden University Medical Center, Leiden, The Netherlands; 6 Department of Neurology, Leiden University Medical Center, Leiden, The Netherlands; 7 Department of Neurology, Danish Headache Center, Copenhagen University Hospital–Rigshospitalet, Copenhagen, Denmark; 8 Danish Knowledge Center on Headache Disorders, Glostrup, Denmark

## Abstract

Migraine aura – manifesting as transient, neurological disturbances – presents a complex and unresolved relationship with migraine headache. Cortical spreading depolarization (SD), recognized as the mechanism underlying aura symptoms, has been shown to trigger head pain through activation of trigeminal nociceptors in animal models. However, recent clinical data challenge the notion that aura causes migraine headache in patients. In this Essay, we critically examine the pathophysiology of migraine aura and migraine headache, exploring evidence from clinical observations, (genetic) mouse models, and pharmacological studies. We also discuss the role of SD, the trigeminovascular system, and the impact of pharmacological agents that both trigger and treat migraine attacks. Our essay highlights the complexities and conflicting data surrounding the interplay between aura and headache, emphasizing the need for further research to unravel this mystery and improve therapeutic strategies for individuals with migraine.

## Abstract 2

The relationship between migraine aura and headache has been the subject of a long-standing debate. With insights from clinical, pharmaceutical, and in vivo animal studies, this Essay delves into the mechanistic and pathophysiological links between migraine aura and headache.

## Introduction

Migraine aura is a captivating phenomenon in neurology that intrigues both clinicians and neuroscientists due to its striking clinical manifestations and complex pathophysiology. Characterized by reversible neurological disturbances that often precede the headache phase, migraine aura offers a unique window into the underpinnings of migraine – a prevalent and disabling disorder – and into the functional dynamics of the human brain. The link between migraine aura and headache has been the subject of long-standing debate, with the causal mechanisms underlying this relationship remaining unresolved.

The prevailing hypothesis implicates spreading depolarization (SD), a transient pathophysiological process of the brain responsible for aura symptoms, as a potential instigator of events culminating in migraine headache, at least in a subset of patients. This notion is supported by preclinical studies demonstrating the activation of trigeminal nociceptive neurons following the initiation of cortical SD. However, new perspectives on this mystery emerge from studies of pharmacological agents known to trigger migraine headache. These substances are believed to initiate migraine via activation of the trigeminovascular system, a term describing the sensory network originating from the trigeminal ganglion, innervating intracranial blood vessels and the meninges [1,2]. Notably, although these agents mostly act outside the central nervous system (CNS), they can still induce aura symptoms in some patients. Furthermore, monoclonal antibodies targeting the calcitonin gene-related peptide (CGRP) pathway – large molecules that are unlikely to enter the CNS – have demonstrated efficacy in both migraine with and without aura [3]. These observations suggest a common peripheral mechanism as the initiating event of migraine attacks in both with and without aura – a mechanism that could possibly be a trigger of migraine headache independently of SD.

Increasing our understanding of the migraine-aura relationship is clinically relevant since interventions targeting SD will only prevent migraine headache if SD is indeed a causative factor. In this essay, we delve into the current understanding of the complex interplay between migraine aura and headache by examining the pathophysiological processes, genetic insights, and therapeutic approaches.

## Clinical features of migraine aura and migraine headache

Migraine with aura is characterized, per definition, by recurrent episodes of fully reversible CNS symptoms [4]. Aura episodes typically last for less than 1 h before resolving. Visual disturbances are the most common manifestation, occurring in more than 90% of migraine with aura attacks. Patients may experience neurological symptoms other than visual, either in combination or as the sole clinical manifestation, and these symptoms may vary from one attack to another. Apart from visual symptoms, somatosensory and speech and/or language symptoms are also common, reported by roughly one-third to one-fifth of migraine with aura patients, respectively [5–7]. Patients typically experience symptoms in one half of the visual field or one side of the body, reflecting the origin of the symptoms in the contralateral cerebral hemisphere. A characteristic clinical feature of migraine aura is the gradual spread of symptoms, usually over 5–20 min for each symptom. In addition, migraine aura symptoms are often positive phenomena, i.e., patients may experience visual phenomena such as flickering, geometrical shapes, and colors; and skin sensations like prickling or “pins and needles” sensations during aura episodes. Patients experiencing visual aura often report being able to perceive these positive features with their eyes closed. Notably, individuals who are blind due to disorders of the eye or optic nerve may also experience visual auras [8]. In contrast, negative symptoms, such as loss of sensory function, can also occur during migraine aura, typically following positive symptoms. A classical visual aura is exemplified by a flickering zig-zag line near the center of the visual field, gradually spreading towards the periphery on one side over several minutes, leaving a scotoma (blind spot) in its wake.

Rarely, patients experience motor symptoms during migraine aura episodes. This is considered a separate diagnostic entity called hemiplegic migraine, which has a prevalence of only 0.01% [9]. Hemiplegic migraine may be either familial (when at least one first- or second-degree relative has the same phenotype) or sporadic; the two forms being equally prevalent [10]. Motor aura symptoms are always negative, manifesting as motor weakness rather than involuntary movement. Attacks of hemiplegic migraine may have other, more severe, clinical features that are not seen in typical migraine aura attacks, including impaired consciousness, leading to coma, fever, or meningism [11]. While patients nearly always recover fully, symptoms may last for days to several weeks, and cases of fatal brain edema resulting from severe hemiplegic migraine attacks have been reported [12].

### Clinical relationship to headache

Co-occurrence of migraine aura and migraine headache varies considerably between patients and can also vary between attacks in the individual patient. As a whole, migraine attacks most commonly present without aura symptoms, characterized by episodes of unilateral, pulsating headache associated with hypersensitivity to light (photophobia) and sound (phonophobia), as well as nausea and vomiting. Migraine without aura, the diagnostic entity characterized by these types of attacks without aura symptoms, is approximately twice as common as migraine with aura [13]. Migraine aura symptoms can also occur without an associated headache. In individuals who experience both aura and headache during their migraine attacks, aura symptoms may present before, during or after the onset of headache. The same patient may experience migraine attacks with and without aura. Studies of co-occurrence of migraine without aura in individuals diagnosed with migraine with aura have provided varying results, with co-occurrence rates from 13% to 89% [7,14,15].

Considering the temporal relationship between aura and headache is essential to determine the causal connection between the two phenomena. While it is generally observed in clinical practice that migraine aura symptoms occur before the onset of headache, only a few studies have systematically investigated the relationship. An overview of these studies and their main findings is provided in Table 1.

**Table 1:** Clinical studies providing information about the temporal relation between aura and headache symptoms in patients with migraine with aura.

Study	Design and study population	Findings
Russell and Olesen [5].	A semi-structured headache interview study of 4,000 people from the general population.	Of 163 individuals with migraine with aura, headache followed aura in 92%, aura and headache occurred simultaneously in 5%, and aura followed headache in 3%.
Hansen et al. [16].	A prospective study of 267 patients with migraine with aura, involving 16 headache centers.	73% of patients reported headache during the aura phase and 61% within the first 15 min after aura onset. Other migraine symptoms frequently reported during the aura were nausea (51%), photophobia (88%), and phonophobia (73%). Within the first 15 min of aura onset, 54% of patients reported headache fulfilling the criteria for migraine.
Viana et al. [14].	A diary-aided study of detailed recordings of aura symptoms from 54 migraine with aura patients from two tertiary headache centers.	The headache phase started after the end of aura in 36% of patients, simultaneously with the end of aura in 15%, during the aura in 26%, simultaneously with the onset of aura in 14%, and before the aura in 9%.
Thomsen et al. [7].	A semi-structured headache interview study of 227 migraine with aura patients from a tertiary headache center.	81% of participants experienced onset of headache after the onset of aura, with a mean delay of 42 min, while 12% reported simultaneous onset of aura and headache. 5% reported aura onset after headache onset.

While attacks of headache preceding aura symptoms have consistently been reported, this does not entirely rule out SD as the headache-triggering mechanism even in these attacks, since initial symptoms of SD can be subtle. Further, in such attacks, SD could theoretically arise in non-eloquent cortex and propagate from there into cortical areas where it gives rise to aura symptoms, causing a delay from actual SD onset to clinical aura.

Migraine aura without headache is occasionally experienced in around 40% of individuals with migraine with aura [5]. The true prevalence of migraine aura without headache, however, is still unknown. Epidemiological studies aimed at determining migraine prevalence have used initial questions of either “have you ever had a headache?”, “have you ever had migraine?”, or “have you ever had visual disturbances lasting from 5 to 60 min, which were followed by a headache?”. These questions may not capture patients who exclusively experience migraine aura symptoms without headache. In a survey among Norwegian neurologists, 83 out of 245 responders (34%) reported having experienced migraine aura without headache at least once, suggesting that this condition may be underdiagnosed.

### Premonitory symptoms

**Fig 1:**
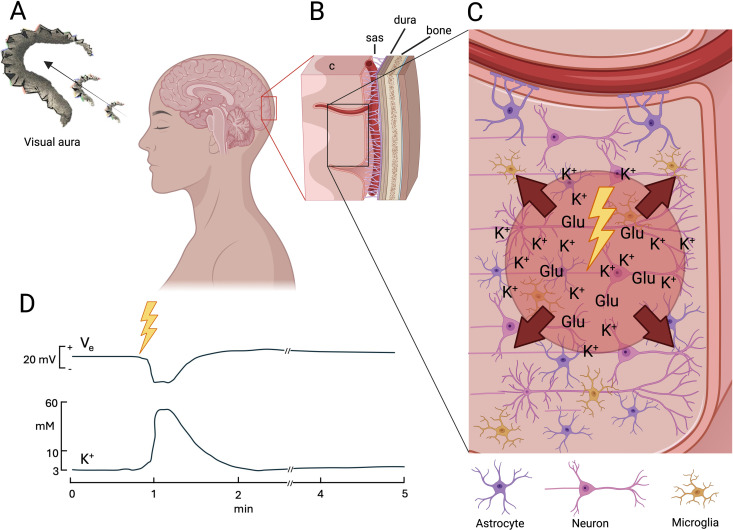
Initiation and propagation of spreading depolarization. **(A)** A visual migraine aura in the form of a gradually developing scintillating scotoma (adopted from Airy, Phil. Trans. R. Soc. 1870) [41], reflecting a spreading depolarization (SD) wave propagating in the visual cortex. **(B)** Occipital cortex (c) and its anatomical relation to the subarachnoid space (sas) with pial blood vessels, the meninges, and the skull. **(C)** Close-up view of the cerebral cortex, containing neurons and glial cells (only astrocytes and microglia depicted here). The initiation of SD in migraine is still poorly understood but it is likely that a combination of strong excitatory activity, reduced inhibitory activity, and/or reduced clearance of excitatory substances, perhaps in combination with metabolic compromise, is required to simultaneously depolarize a minimum critical volume of gray matter (lightning bolt), raising the extracellular [K^+^] to above approximately 12 mM. Recent work shows that along with K^+^ release, there are large glutamate (Glu) release events that likely interact with [K^+^] elevation to form a nidus of SD initiation. Mechanisms that help buffer extracellular [K^+^] and excitatory neurotransmitters, such as astrocytic uptake of K^+^ and glutamate, limit SD initiation and propagation. When a critical threshold is reached, neuronal membrane resistance is rapidly lost due to a large cation conductance, resulting in a massive K^+^ efflux raising the extracellular K^+^ concentration to 20–60 mM. Meanwhile, influx of Na^+^, Ca^2+^, and water leads to marked cell swelling and release of neurotransmitters, including Glu, to the extracellular space. The resulting increased extracellular [K^+^] and concentration of excitatory neurotransmitter triggers the same reaction in neighboring neurons and glial cells. In this way, SD spreads as a wave of tissue depolarization traveling at a slow rate of 2–5 mm/min through contiguous gray matter. **(D)** The depolarizing wave and associated K^+^ efflux is accompanied by a dramatic change of the local electrical potential (*V*_*e*_). In otherwise normal brains, the disturbance is self-limiting and apparently harmless, and after a few minutes, normal cellular ion homeostasis is re-established, though other parameters such as neurovascular coupling can take over an hour to return to normal. Note the logarithmic scale. Adapted from Hansen and Zeuthen (Acta Physiol Scand, 1981) [35]. Created with Biorender.

Premonitory (or prodromal) symptoms of migraine are a diverse range of non-headache symptoms that may appear hours to even days before the headache phase in migraine without aura or before the aura phase in migraine with aura, acting as early warning signs (see Fig 1). Typical symptoms include irritability, fatigue, drowsiness, emotional changes, yawning, and food cravings. The proportion of individuals with migraine who experience premonitory symptoms varies tremendously with estimates ranging from 8% to 100%, depending on the study population (general population or clinic-based sample) and assessment method [17].

Based on the symptomatology and functional neuroimaging studies, involvement of brain structures primarily including the hypothalamus, brainstem, and limbic system have been suggested (Fig 1) [18]. Regarding the causal relationship between migraine aura and headache, it is notable that the prevalence of premonitory symptoms is similar in migraine with and without aura, although some studies have found a slightly higher prevalence in migraine with aura [15,17,19–22]. These observations suggest that SD may not be the initiating event in all attacks of migraine with aura and that a common upstream mechanism, possibly originating in diencephalic or limbic structures [2], may be involved in the initiation of migraine with as well as without aura, at least in a subset of patients. More specifically, since SD in migraine without aura has not been demonstrated, such a hypothetical mechanism would trigger migraine headache, and not SD, in attacks of migraine without aura, while in attacks of migraine with aura, it would trigger headache and SD, respectively, via separate pathways, rendering a direct causal relationship between SD and migraine headache unnecessary. Indeed, a recent functional magnetic resonance imaging (MRI) study of a single migraine patient who underwent daily imaging during trigeminal nociceptive stimulation for 30 consecutive days reported increased activation of the hypothalamus preceding one attack of migraine with aura as well as one attack without aura [22]. These data from two migraine attacks in a single subject should be interpreted with considerable caution, and additional studies are needed to confirm these findings. Regarding migraine premonitory symptoms in general, it should be noted that the clinical significance and mechanisms of these symptoms are yet poorly understood and that no firm conclusions concerning the causal relation between migraine aura and migraine headache can be drawn based on studies of premonitory symptoms currently.

## Cortical spreading depolarization and migraine aura

SD is widely recognized as the electrophysiological mechanism underlying migraine aura [23]. Early evidence for this connection comes from the work of Karl Lashley, an American psychologist who meticulously documented his own visual auras. He noted their homonymous presentation and gradual spread across the visual field, concluding that they represented dysfunction in the visual cortex that propagated at a rate of approximately 3 mm/min. This rate closely aligns with the propagation of SD observed in experimental studies involving animal models [24]. Similarly, the typical sensory symptoms associated with migraine aura, such as cheiro-oral symptoms, manifest as a gradual spread from the distal upper extremity to the ipsilateral peri-oral and intra-oral regions. This pattern corresponds precisely to the propagation of SD in the adjacent somatosensory cortical areas of the contralateral hemisphere that represent these body parts [5]. Around the same time as Lashley’s observations, Aristides Leão, a Brazilian physiologist, discovered SD, through electrocorticography (ECoG) *in vivo* studies of rabbits [25]. Leão proposed that the phenomenon serves as the underlying substrate for migraine aura. However, this suggestion garnered little attention until the 1980s. During this period, a series of cerebral perfusion studies performed in patients during migraine aura symptoms demonstrated gradually spreading cortical perfusion changes that matched the propagation of SD [26]. Subsequently, functional MRI data obtained during visual auras [27] and somatosensory auras [28] further corroborated that migraine aura results from SD in the human brain. Very recently, SD was demonstrated by intracranial stereotactic electroencephalographic recording during migraine with aura in a single patient [29].

### Physiology of cortical spreading depolarization

SD is a distinct neurophysiological phenomenon observed across various species, from insects and cephalopods to reptiles, birds and mammals, including non-human primates and humans [30–32]. It is a wave of intense, transient depolarization of neurons and glia cells that propagates in gray matter structures of the CNS. In the context of migraine aura, spreading depolarization primarily affects the cerebral cortex, hence cortical spreading depolarization, CSD. A wave of SD is initiated in an all-or-none fashion as a response to intense cell membrane depolarization. Experimentally, SD can be elicited by electrical, mechanical, and chemical stimuli [33], or through optogenetics [34].

**Fig 2:**
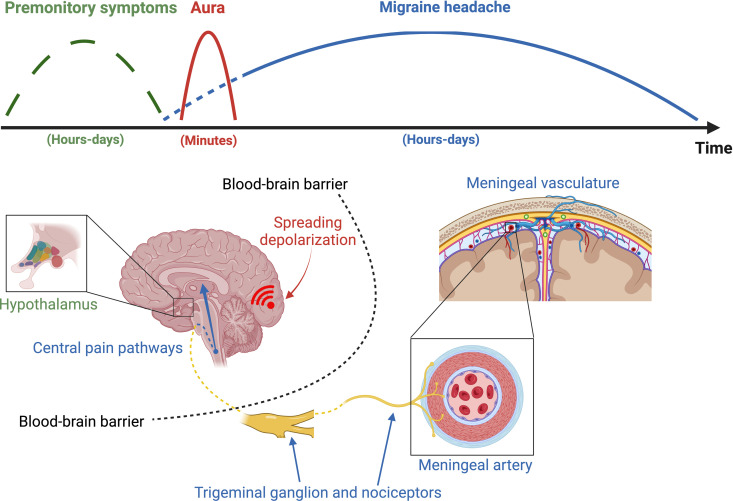
A simplified overview of proposed mechanisms underlying different phases of a migraine attack. Premonitory symptoms (green) – symptoms occurring up to 48 h before headache onset in a subset of patients – have been suggested to arise from the hypothalamus or other diencephalic structures, although their origin is largely unknown. The migraine aura (red), which usually precedes the onset of migraine headache, represents spreading depolarization propagating in eloquent cerebral cortex. Migraine headache (blue) may, in a minority of patients, start before the aura symptoms. The head pain of migraine is believed to depend on sensory input from activated meningeal nociceptors, involving the release of vasoactive peptides and vasodilation. Nociceptive signals project to central pain pathways via the trigeminal ganglion and upper cervical sensory ganglia (the latter not depicted). Note that structures believed to be involved in the premonitory and aura symptoms are protected by the blood–brain barrier, while structures involved in the nociceptive pathway are located inside as well as outside of the blood–brain barrier. Created with Biorender.

A sufficiently strong triggering stimulus causes an increase of the extracellular K^+^ concentration ([K^+^]_e_) above approximately 12 mM [35,36] and depolarizes a critical volume of 0.03 to 1 mm^3^ (at least in the rodent brain) [37,38]. Focal initiation of SD in uninjured brain – as expected in migraine – likely requires voltage-gated Ca^2+^ channel-dependent activation of N-methyl-D-aspartate (NMDA) receptors [39] and involves a sudden and complete loss of membrane potential, and K^+^ efflux leading to a 10- to 20-fold increase of [K^+^]_e_ compared to the resting state. An interplay of K^+^-induced depolarization and glutamate release (which causes further depolarization and K^+^ efflux) is likely involved in bringing the cortex to SD threshold [30–32]. Recently, large glutamate release events, termed glutamate plumes, have been observed prior to the onset of SD, in the region where SD originates [40]. Meanwhile, influx of Na^+^, Ca^2+^, and water leads to marked cell swelling and release of neurotransmitters, including glutamate, to the extracellular space. This reaction may be explained by opening of non-selective large-conductance cation channels, leading to passive transmembrane cation transport along concentration gradients [30–32], although such channels have not yet been identified and this phenotype could also be the result of the opening of multiple channel types. The resulting increased [K^+^]_e_ and excitatory neurotransmitter concentration triggers the same reaction in neighboring neurons and glia cells. In this way, SD spreads as a wave of tissue depolarization traveling at a slow rate of 2–5 mm/min through contiguous gray matter (Fig 2). Depolarized neurons may initially exhibit bursts of action potentials as the wave propagates [42]. Following complete membrane depolarization, action potential firing is no longer possible, and all spontaneous or evoked electrical activity in the affected area is suppressed. Indeed, the slowly propagating pattern of brief neuronal firing is followed by a long-lasting neuronal silence that is the electrophysiological hallmark of SD. The term spreading depression, originally coined by Leão [25], is often used interchangeably with the now more common term “spreading depolarization”. In the wake of the SD wave, homeostasis and transmembrane ion gradients are restored, usually within a minute, through various mechanisms, including the Na^+^-K^+^ ATPase and astrocytic buffering.

Astrocytes are efficient buffers of [K^+^]_e_ and extracellular excitatory neurotransmitters. A high astrocyte density in gray matter tissue will potentially limit SD propagation and increase the threshold for initiation. Also, the short distance between neurons will likely facilitate the spread of SD through extracellular diffusion of K^+^ and glutamate. Consequently, brain areas with a high neuron-to-astrocyte ratio would be expected to have a lower threshold for triggering SD. Early work appeared to agree, with measurements of neuronal density and neuron/glia ratio appearing consistent with this hypothesis [43]. More recent work in both rodents and humans, however, shows that in sensory cortices increased neuronal density is met with increased glial density, such that the neuron/glia ratio is similar to, or in fact lower than, other cortices [44,45]. It is possible that anatomical complexity (e.g., lamination and region-specific specializations) can contribute to SD susceptibility: in mice, the most susceptible region is the barrel cortex, which is no different from other sensory regions in neuron/glia ratio but whose barrels may contribute to altered diffusion [46]. Both the hippocampus and cortex, dendritic layers with complex arborizations, are more likely than other layers to serve as a nidus of SD ignition [47]. Factors that enhance brain excitability or diminish the ability to buffer [K^+^]_e_ and excitatory neurotransmitters (e.g., genetic, hormonal, environmental, pharmacological, and pathological) facilitate the occurrence and propagation of SD. This is borne out in genetic models of migraine, as discussed below.

### Initiation of cortical spreading depolarization in migraine with aura: Insights from genetic mouse models

Genetic mutations that intrinsically change brain activity provide a unique opportunity to investigate mechanisms involved in the initiation and consequences of SD, and thereby in the mechanisms of migraine with aura. A particularly valuable source for such studies are transgenic mouse models that express gene mutations previously identified in patients with rare monogenic forms of migraine. Most progress has been made for familial hemiplegic migraine (FHM), a rare subtype of migraine with aura [4]. Except for a transient hemiparesis during the aura, clinical symptoms of hemiplegic migraine are generally identical to those of typical migraine with aura (see above); of note, attacks can be provoked by similar trigger factors [48] and treated and prevented with largely the same medications [49]. There are three genes that are undisputed in causing FHM: *CACNA1A* (FHM type 1, FHM1), *ATP1A2* (FHM2), and *SCN1A* (FHM3)), which all encode ion transporters involved in controlling synaptic activity. Transgenic knock-in (KI) mouse models that express missense mutations in the mouse ortholog genes were all shown to exhibit brain hyperexcitability as a result of increased local extracellular K^+^ and glutamate in the cortex [50,51], which are strong facilitators of SD [32]. FHM mutant mouse models revealed an enhanced susceptibility to experimentally induced SD (be it by local application of K^+^, electrical stimulation, or optogenetics) brought about by increased excitatory neurotransmission either due to hyperactive neuronal voltage-gated Ca_V_2.1 Ca^2+^ channels (in FHM1) [50], by reduced glial uptake of K^+^ and glutamate via defective α2 Na^+^-K^+^ ATPases (in FHM2) [40,52–54], or hyperactive neuronal voltage-gated Na_V_1.1 Na^+^ channels (in FHM3) [55,56]. Moreover, increased presynaptic excitability upon repetitive stimulation was also shown for KI mice that express a missense mutation in casein kinase 1 delta (CK1δ), previously identified in a family with patients suffering from migraine with aura and advanced sleep phase syndrome [57,58]. Here, a reduction in presynaptic adaptation at excitatory (and not inhibitory) synapses and increased glutamatergic transmission was observed in cortical brain slices.

Of special interest, also for future studies into the initiation mechanisms of SD, is the recent observation that such events can occur spontaneously – in the absence of cortical damage caused by the induction method – in FHM1, FHM2, and FHM3 mutant mice [55,59,60]. Although in FHM1 mutant mice spontaneous SD events were very rare (only six events in 140 days of ECoG recording with invasive electrodes), they were much more frequent in FHM3 mutant mice (three events in six days of ECoG recording with epicranial electrodes). Recently, spontaneous SD (on average one per day during 7 days of recording brain activity) have been reported for a novel FHM2 mutant mice [60]. In addition, ablation of α2 Na^+^-K^+^ ATPase activity using conditional *Atp1a2* mice also resulted in spontaneous SD events that correlated with transient motor impairment [61]. Spontaneous SDs in FHM1 mutant mice were associated with body stretching, one-directional slow head turning, and rotating movement of the body. Notably, similar movements as well as a reduced contralateral unilateral forepaw grip performance (that could not be assessed for the spontaneous events) were seen when SDs were induced through optogenetics [59], which also provides at least some first validity that head pain may occur after spontaneous SD. A study by Dehghani et al. [62] showed that optogenetically induced SDs resulted in various head pain phenotypes (measured as increased mouse grimace scale (MGS) scores) [63] and the occurrence of oculotemporal strokes at 30 min, which had normalized in wildtype mice at 24 h and in mutant mice only at 48 h. In addition, the FHM1 mutant mice showed prolonged brain neuroinflammation compared to wildtype mice. A link between SD and a head pain phenotype was also shown in a study in which multiple optogenetically induced SD events were triggered in wildtype mice that elicited periorbital mechanical allodynia (i.e., pain caused by a stimulus that does not normally elicit pain), interpreted as trigeminal pain, increased MGS scores, and anxiety behavior [64]. Finally, a more mechanistic link between an FHM mutation and the induction of a pain phenotype was shown for FHM2 mutant mice; administration of nitroglycerin, used also in migraine patients to elicit migraine-like attacks, resulted in orofacial mechanical hypersensitivity, indicative of migraine-like cranial pain, through changed neuronal activity in the cingulate cortex, a region that is crucial for pain processing [65].

## The trigeminovascular system and migraine headache

The head pain experienced during a migraine headache is typically believed to originate from nociceptors of the trigeminal nerve that innervate the meninges (pia, arachnoid, and dura) as well as large cerebral arteries and sinuses (Fig 2), thus serving as an example of a visceral pain syndrome, although without evidence of tissue injury or inflammation, with referred pain [66]. Pain induced by electrical or mechanical stimulation of these structures during brain surgery in conscious patients [67], as well as pain associated with meningitis [68], is remarkably similar to migraine headache, featuring the characteristic throbbing pain, nausea, vomiting, photophobia, and phonophobia. The meningeal vasculature is densely innervated by nociceptive unmyelinated C-fibers and thinly myelinated A*δ* fibers, primarily originating from the ophthalmic (V1) division of the trigeminal nerve [1,2]. These sensory nerve fibers contain peptides with potent vasodilatory effects, such as substance P, calcitonin gene-related peptide (CGRP), and pituitary adenylate cyclase-activating polypeptide (PACAP). The latter two peptides are established targets for anti-migraine pharmacotherapy [69,70], highlighting their critical role in the generation of migraine pain.

This network of vasoactive neuropeptide-containing projections from trigeminal sensory nerve fibers to the meninges and its associated blood vessels is commonly referred to as the trigeminovascular system, a term coined by Moskowitz and colleagues in 1979 [71]. Because the anatomical regions responding to pain in migraine extend to C2, and because the phenotypes of migraine often involve the neck as well as the head, the term’s meaning has broadened with time to include not just the trigeminal nerve and nucleus caudalis but the closely associated C1 and C2 nerves, which collectively with the trigeminal nucleus caudalis form the trigeminocervical complex [1,2]. Because more recent research has shown the involvement of a much broader neural network, the concept has also extended beyond peptidergic C-fibers to include other sensory neuron types, and even beyond sensory nerves to include autonomic efferents involved in headache phenotypes. The mechanisms leading to the activation of the trigeminovascular system during migraine attacks remain unclear but likely involve the initial release of vasoactive peptides from peripheral trigeminal vascular neurons, which may further activate sensory neurons. For instance, CGRP released from C-fiber terminals can activate adjacent A*δ* nerve terminals [72]. Once activated, peripheral trigeminovascular neurons become sensitized to stimuli, meaning that their response threshold decreases and the magnitude of their response increases [73]. As for the root cause of this peptide release, one hypothesis is that the initial event leading to migraine headache originates in the hypothalamus, which through activation of a parasympathetic pathway involving the superior salivatory nucleus and the sphenopalatine ganglion, leads to the release of acetylcholine and other substances causing intracranial vasodilation and meningeal nociceptor activation [73]. There are likely multiple other pathways to craniocervical nociceptor activation, as suggested by the ability of compounds that have poor CNS penetration to provoke migraine attacks.

The trigeminal nociceptors are pseudo-unipolar neurons with cell bodies located in the trigeminal ganglion and axons that project to both peripheral and central sites. Afferent projections from the trigeminal ganglion converge with inputs from C1 to C2 innervated tissues (including pericranial and paraspinal muscles) and synapse with second-order neurons in the trigeminocervical complex, which consists of the trigeminal nucleus caudalis and the dorsal horn of the upper cervical spinal cord (C1–C2) [74]. From the trigeminocervical complex, ascending pathways project to brainstem, thalamic, hypothalamic, and basal ganglia nuclei, and from there to various cortical areas involved in processing nociceptive signals [1,2]. Sensitization of central trigeminovascular neurons in the trigeminocervical complex and thalamic nuclei is thought to be responsible for cephalic allodynia, the perception of pain due to normally innocuous stimuli, such as light touch on the skin of the head or the cephalic muscles [75,76].

In summary, the trigeminovascular system is central to the pathophysiology of migraine, linking peripheral meningeal nociception to central pain pathways via a complex network of sensory and autonomic fibers. However, the precise mechanisms initiating the release of vasoactive peptides such as CGRP and PACAP remain unclear. Furthermore, it is not yet understood how these peripheral events interact with central sensitization processes to produce the full migraine phenotype, including cephalic allodynia.

## Preclinical studies linking SD and headache mechanisms

Several lines of experimental evidence provide a biological substrate linking SD to trigeminal nociception. For example, SD induces periorbital allodynia in rodents [64,77–80], mimicking the allodynia experienced by individuals with migraine [76,81]. Trigeminal-specific allodynia in mice develops within an hour after a single cortical SD and resolves within two days, whereas repeated SDs result in more severe and longer-lasting allodynia [64]. Notably, SD-induced allodynia is blocked by sumatriptan, a 5HT_1B/1D_ receptor agonist used as selective migraine abortive treatment, and shows sexual dimorphism associated with the estrus cycle, in line with the higher prevalence of migraine in females [64,80,82,83].

SD also activates primary meningeal trigeminal afferents and second-order neurons in the trigeminal nucleus caudalis with a temporal pattern that might be consistent with headache after aura [84–86]. SD also augments meningeal mechanosensitivity, potentially explaining how maneuvers that transiently increase intracranial pressure can exacerbate migraine headache [87]. Furthermore, CGRP antagonism inhibits SD-induced nociceptive activation and sensitization of brainstem neurons [84,88–90]. Given the clinical efficacy of CGRP antagonists in migraine prevention, these findings suggest shared mechanisms between SD-induced trigeminal pain and migraine headache.

Elsewhere, cortical SD evokes meningeal vasodilation, upregulation of proinflammatory cytokines, and plasma protein leakage, which are possible signs of neurogenic inflammation, in some cases abrogated by trigeminal transection [91–93]. In addition, SD induces complex changes in dural immune cells, such as macrophages and dendritic cells, that are consistent with an inflammatory response; once again, these changes show a temporal pattern that seems consistent with aura-induced headache [94]. Indeed, PET imaging in humans using translocator protein receptor-ligand targeting immune cells revealed interictal meningeal and para-meningeal inflammation [95]. Supporting these findings, SD induces rapid neuroinflammation and microglial activation [96–105], although the relevance of the inflammatory changes in the brain parenchyma for trigeminal nociception remains unclear [106]. Notwithstanding, exactly how SD activates nociception in these models is yet unresolved. It is thought that the multitude of substances released from the parenchyma during and in the wake of SD (e.g., K^+^, nitric oxide, arachidonic acid metabolites, amino acids, cytokines, and peptides) reach the meninges via diffusion or bulk cerebrospinal fluid flow and activate or sensitize trigeminal nerve endings innervating the meninges [107,108]. Still, an MRI study found no association between headache occurrence following aura and the distance from cortical surface to the overlying dura in patients, challenging the notion of straightforward meningeal nociceptor activation caused by substances released from the cortex [109]. Recent studies, however, suggest that substances released during CSD may reach the meninges via more specific anatomical pathways. In particular, structures such as the arachnoid cuff [110] and dural lymphatics [111,112] have been identified as direct links between the cortex and dura. Furthermore, these pathways may facilitate the transport of nociceptive mediators to the trigeminal ganglion via cerebrospinal fluid drainage routes [113]. This emerging evidence expands our understanding of the mechanisms by which cortical events may trigger migraine.

## Pharmacological insights

### Human migraine provocation models

A hallmark of migraine is its susceptibility to being triggered by a range of factors. This characteristic allows for the experimental induction of migraine attacks, providing an opportunity to investigate patients during attacks and to study the underlying mechanisms of migraine [114]. Several chemicals, including endogenous signaling molecules and pharmaceuticals, have been shown to trigger migraine attacks. In addition, experimental exposure to normobaric hypoxia induces migraine [115,116]. Notably, all substances known to induce migraine, as well as normobaric hypoxia, cause dilation of cranial arteries. Human migraine provocation studies most commonly use a randomized, double-blind, crossover design where patients with migraine and healthy volunteers are allocated to receive an active, putative trigger drug, an intervention, a placebo or a sham treatment. Following provocation, headache occurrence, characteristics, and accompanying symptoms are recorded. The studies have consistently shown that only individuals with a history of migraine can have migraine attacks provoked, whereas healthy volunteers may develop a mild headache, but not migraine [114].

Currently established molecules with migraine-triggering abilities include the nitric oxide donor, glyceryl trinitrate, CGRP, PACAP, cilostazol (a phosphodiesterase-3 inhibitor), sildenafil (a phosphodiesterase-5 inhibitor), an adenosine triphosphate-sensitive potassium (K_ATP_) channel opener, and a large conductance calcium-activated potassium (BK_Ca_) channel opener [117]. These substances exert effects on vascular smooth muscle cells that ultimately lead to opening of potassium channels and vasodilation. The exact mechanisms by which these reactions lead to the full clinical spectrum of migraine symptoms – including unilateral throbbing headache, photophobia, and nausea – remain not fully understood, particularly why this full response manifests only in patients with migraine. It has been hypothesized that the combination of potassium release from vascular smooth cells and vasodilation sensitizes perivascular trigeminal afferents, ultimately causing the perception of pain; however, experimental evidence supporting this mechanism is lacking [118]. Several migraine-provoking compounds have been shown to induce cutaneous hypersensitivity or light-avoidance in behavioral *in vivo* mouse models [119]. Human provocation studies have also contributed to the development of anti-migraine drugs that specifically target trigger molecules, most notably in the case of CGRP, with several currently approved drugs [120]. Most recently, a humanized monoclonal antibody targeting PACAP-38 has shown efficacy in patients with migraine [70].

### Experimental induction of migraine with aura

Although migraine without aura is the most prevalent migraine subtype, several studies of migraine provocation have included patients with migraine with aura. An overview of these studies is provided in Table 2.

**Table 2:** Experimental migraine provocation studies including migraine patients with aura.

Study	Study population	Provocation method	Outcome
Christiansen et al. [121]	12 migraine with aura patients, who had never previously experienced migraine without aura attacks.	Intravenous infusion of GTN.	None of the patients reported migraine aura following provocation but six experienced a first-ever migraine attack that did not include an aura.
Sances et al. [122]	Patients with primary headache disorders, including 22 patients with migraine with aura (of whom five also had a history of migraine without aura).	Sublingual administration of GTN.	Three of the 22 patients developed their usual aura symptoms, and another six reported migraine without aura.
Hansen et al. [123]	14 migraine with aura patients, who had never previously experienced migraine without aura attacks.	Intravenous infusion of CGRP.	Four of the 14 patients developed their usual aura symptoms followed by headache, and a further five fulfilled criteria for migraine without aura.
Hougaard et al. [124]	27 migraine with aura patients (14 also had a history of migraine without aura).	Strenuous physical activity and photostimulation	Three patients reported aura (one after exercise only, two after exercise combined with photostimulation). All experienced headache as well. A further three reported migraine without aura following exercise.
Arngrim et al. [115]	15 migraine with aura patients, who had never previously experienced migraine without aura attacks.	Normobaric hypoxia and sham exposure in a cross-over design.	Hypoxia induced aura in three patients, all of whom experienced headache as well. No patients reported aura after sham. In total, hypoxia induced migraine attacks in eight patients compared to one patient after sham.
Butt et al. [125]	16 migraine with aura patients (four also had a history of migraine without aura).	Oral administration of cilostazol and placebo in a cross-over design.	One patient reported aura after cilostazol, and one after placebo. Headache in 12 patients after cilostazol and in one patient after placebo fulfilled criteria for migraine without aura.
Frank et al. [116]	16 migraine with aura patients (prevalence of concomitant migraine without aura not reported). Fourteen patients with migraine without aura.	Normobaric hypoxia exposure.	Three out of the 16 individuals with migraine with aura, as well as two of 14 initially diagnosed with migraine without aura, developed aura following provocation. All patients reporting aura, experienced headache as well.
Hougaard et al. [126]	14 migraine with aura patients (four also had a history of migraine without aura)	Intravenous infusion of endotheln-1 and placebo in a cross-over design.	No patients reported migraine aura or migraine headache following endothelin-1 infusion.
Karsan et al. [127]	53 migraine patients (27 also had a history of migraine without aura).	Intravenous infusion of GTN.	Seven of the 53 provoked migraine patients reported development of aura symptoms (the triggering rate specifically in the subgroup of migraine with aura patients was not reported). Four patients reported the same symptoms again on a second GTN exposure.
Al-Karagholi et al. [128]	17 migraine with aura patients, who had never previously experienced migraine without aura attacks.	Intravenous infusion of levcromakalim and placebo in a cross-over design.	Aura was reported by 10 of the 17 patients following levcromakalim. All 10 patients reported headache as well. No patients reported aura after placebo. An additional four patients reported migraine attacks without aura after levcromakalim.
Butt et al. [129]	16 migraine with aura patients (five also had a history of migraine without aura).	Oral administration of sildenafil and placebo in a cross-over design.	Sildenafil induced aura symptoms in three out of 16 patients. All three patients reported migraine headache after aura. A further six reported migraine without aura. No patients reported aura symptoms after placebo administration.
Al-Khazali et al. [118]	34 migraine with aura patients (29 also had a history of migraine without aura).	Intravenous infusion of CGRP.	13 of the 34 patients reported their usual aura symptoms following infusion. All 13 patients reported headache as well. In total, 24 patients reported migraine headache.
Thomsen et al. [130]	27 migraine with aura patients (10 also had a history of migraine without aura).	Intravenous infusion of levcromakalim and placebo in a cross-over design.	Four participants experienced migraine attacks with aura following levcromakalim compared to two after placebo. All patients reported headache as well. An additional 10 patients reported migraine attacks without aura after levcromakalim.

GTN: glyceryl trinitrate. CGRP: calcitonin gene-related peptide.

Collectively, potent vasodilator substances with different modes of action as well as exposure to normobaric hypoxia, which also causes cranial vasodilation, can trigger migraine aura as well as migraine headache. Conversely, endothelin-1, a potent vasoconstrictor peptide, does not trigger migraine aura or migraine headache in patients [126]. CGRP, one of the most potent aura-triggering agents, is unlikely to cross the blood–brain barrier due to its large molecular weight of 3,790 g/mol [131]. The triggering potential of these vasodilators is clearly higher for migraine headache than for migraine aura. Notably, the timing of aura relative to headache in provoked attacks is similar to that of the patients’ spontaneous attacks with aura, suggesting that the effects of the provoking agents mimic the intrinsic “natural” mechanisms of migraine attack initiation. It is relevant to note that none of the patients reported aura without migraine headache in any of the migraine provocation studies.

### Effects of anti-CGRP monoclonal antibodies in migraine with aura

Four monoclonal antibodies (mAbs) targeting CGRP or its receptor have demonstrated efficacy for the prevention of migraine and are currently marketed [120]. Anti-CGRP-mAbs are interesting tools in terms of determining the causal relationship between migraine aura and migraine headache since they most likely act exclusively outside of the blood–brain barrier. Their anti-migraine effects are therefore believed to be exerted through peripheral mechanisms that block activation of the trigeminovascular system, and not through effects on centrally occurring SD. In support, the mAb fremanezumab does not prevent SD occurrence, in spite of a compromised blood–brain barrier [132], and does not affect SD-induced arterial dilation and plasma protein extravasation in a rat model [88]. Thus, if SD is the primary event in a migraine attack, subsequently leading to migraine headache, individuals with migraine with aura who are treated with anti-CGRP-mAbs would, at least theoretically, continue to experience aura episodes at their usual frequency. In contrast, the headache phase of migraine would be lessened or prevented. Clinical trials of anti-CGRP-mAbs thus far included mixed groups of patients with and without aura, and only a few investigated specifically the effects of anti-CGRP-mAbs on aura symptoms. A *post hoc* analysis of four randomized clinical trials of erenumab (N = 2,682 patients randomized in total) reported efficacy for the prevention of migraine, specifically in the subgroup of patients with a history of aura [125]. No clear difference was observed for treatment responses in subgroups with and without a history of migraine with aura. Admittedly, the study has limitations, mainly because of the small number of patients available for analysis [3]. Meanwhile, *post hoc* analyses of two trials of eptinezumab concluded that the efficacy of migraine prevention for this drug in patients with self-reported aura was comparable to that in the overall study population of patients with and without aura [133]. Of note, 50% of patients reported a history of migraine aura, which is higher than the one-third that is generally observed in migraine populations. The higher aura prevalence in that study may reflect misclassified individuals as migraine with aura patients, likely based on the presence of premonitory symptoms such as (non-aura) visual symptoms.

Since their marketing in 2018, the general clinical experience with anti-CGRP-mAbs is that they prevent migraine attacks with and without aura rather than showing a specific preventive effect for migraine headache only. A number of small case series of migraine with aura patients treated with anti-CGRP-mAbs generally reported a reduction of migraine headache as well as migraine aura frequency [134–138]. However, in one series of 12 migraine with aura patients, a greater reduction of headache relative to aura frequency, and onset of aura without headache in three cases, was reported [139].

### Pharmacological blockade of migraine aura and SD

Currently, there are no established treatment options specifically targeting migraine with aura. Research using animal models has shown that the threshold for the initiation and spread of SD can be influenced. For instance, chronic administration of common migraine (and epilepsy) preventive medications, such as topiramate, valproate, propranolol, amitriptyline, and methysergide, increases the threshold for experimentally induced SD in rats [140]. Of note, these drugs are effective in preventing both migraine with and without aura, questioning if their impact on SD is clinically relevant. NMDA receptor antagonists, such as ketamine, have been shown to inhibit the initiation and spread of SD in both animal and human studies [141]. For instance, a randomized, double-blind trial using intranasal ketamine, with midazolam as active control, demonstrated a reduction in the severity of aura symptoms but did not affect aura duration in patients experiencing prolonged typical migraine aura (lasting between 1 h and 7 days); no conclusions on headache effects were drawn due to limited data [142]. Amiloride, a blocker of epithelial sodium channels, was reported to inhibit pin-prick-induced SD (i.e., the rate of successful trials of SD elicitation following needle insertion into the cortex), but not K^+^-induced SD (i.e., the rate of SD episodes induced by topical K^+^ application to the cortex) in rats and, in an open-label study, reduced aura and headache symptoms in 4 out of 7 patients with otherwise treatment refractory migraine with aura [143]. Tonabersat, a gap junction modulator that blocks SD in animal studies, was initially tested for migraine prevention in trials that mostly included migraine without aura patients and showed no efficacy [144]. A subsequent clinical trial focusing exclusively on migraine with aura patients reported a reduction in the frequency of aura attacks; however, tonabersat did not impact the number of monthly migraine headache days when compared to placebo [145]. Although this suggests that tonabersat might have a specific effect on migraine aura, it currently is not on the market.

## What is the causal relationship between aura and headache?

Animal studies have consistently demonstrated meningeal nociceptor activation and behavioral changes indicative of facial allodynia following triggered SD events (see above). Since migraine aura symptoms most often precede the headache phase and are due to SD, it is logical to assume that SD may trigger subsequent mechanisms underlying the generation of head pain during attacks in migraine with aura (Fig 2). However, as discussed in this Essay, the proposed causal relationship is not straightforward and in fact challenged by several observations in patients: (i) Premonitory symptoms, preceding aura and headache, are frequently reported in migraine with and without aura, suggesting that SD is not the initial cause; (ii) Headache starts before or simultaneously with the onset of aura in a subset of patients; (iii) Migraine-provoking substances, including CGRP (which is unlikely to cross the blood–brain barrier) can trigger migraine aura as well as migraine headache; And (iv) monoclonal antibodies targeting CGRP or its receptor (which are even more unlikely to enter the CNS) effectively prevent migraine aura and headache in patients.

The following, admittedly highly speculative, chain of events would be consistent with these observations above: Migraine is characterized by abnormal activation of the trigeminovascular system, where an initial event—possibly involving parasympathetically mediated dilation of meningeal blood vessels—triggers the release of vasoactive neuropeptides. These neuropeptides activate meningeal nociceptors, leading to the perception of throbbing head pain originating from the meninges, accompanied by symptoms such as nausea, photophobia, and phonophobia. This abnormal behavior of the trigeminovascular system is specific to individuals predisposed to migraine attacks. In these individuals, provocation with vasodilator substances can trigger the same pathological response and result in a migraine attack. However, in people without this predisposition, who do not experience spontaneous migraines, administration of these substances does not induce migraine headache. In some instances, the activation of the trigeminovascular system initiates SD through an unknown mechanism. This process likely involves depolarization of cortical neurons, possibly triggered by the release of potassium from dilating cortical arterioles into the extracellular space in individuals predisposed to migraine aura (e.g., due to less efficient potassium clearance mechanisms). The occurrence of aura is more frequent in spontaneous migraine attacks compared to experimentally induced ones, suggesting that the intrinsic mechanisms of migraine initiation are more effective at inducing SD than current experimental triggers. This may be due to a more direct effect on nerve fibers or blood vessels in close proximity to the cortex.

To counter these arguments, it should be noted that even if this hypothesis holds true, other mechanisms, including SD, may trigger migraine headache as well, and headache-triggering pathways may vary between and within individuals who experience migraine attacks. For example, the mechanisms of premonitory symptoms in migraine are largely unknown, and even if SD is not the first event in the cascade of a migraine attack, it could still be what triggers migraine headache. In addition, headache perceived before aura in a minority of patients could be explained by subtle initial aura symptoms going unnoticed or SD propagating from non-eloquent to eloquent cortex. Finally, migraine-provoking drugs, even if acting in the periphery could trigger central excitation and SD through canonical sensory (e.g., visual, somatosensory) pathways. GTN infusion in patients with a history of migraine with aura may reduce occipital perfusion in the absence of aura symptoms or headache [146]. This indicates that migraine-triggering drugs may provoke changes in relevant cortex, which could potentially be linked to SD initiation. How this relates to the trigeminovascular system remains uncertain, considering that the initial headache following GTN infusion suggests that the system is already activated. Anti-CGRP monoclonal antibodies cross the blood–brain barrier to some extent [147] and may act centrally, or the peripheral effects of these drugs may cause central modulation that prevents SD initiation.

## Conclusions and future directions

The mystery of the mechanisms linking migraine aura and migraine headache should ideally be solved through clinical experimentation. Developing techniques that can specifically trigger or inhibit SD in humans would be key. For instance, inducing SD through targeted, non-invasive transcranial cortical stimulation, which does not affect pain-sensitive intracranial structures, could determine whether SD initiates headache. Similarly, an intervention capable of selectively aborting a SD event without any nociceptive effects would be invaluable. If such an intervention is administered to an individual who in the past consistently experienced headaches following aura, and it successfully resolves aura symptoms while preventing the onset of headache, it would provide compelling evidence that SD is the underlying cause of migraine headache.

In conclusion, despite significant progress in understanding the mechanisms underlying migraine, the precise link between aura and headache remains elusive. Future clinical and experimental research aimed at directly investigating SD and its relationship with migraine pain remains crucial. Especially, the development of novel techniques to specifically manipulate SD in humans holds great promise for unraveling the “migraine aura mystery”. Such advancements would not only deepen our knowledge of migraine pathophysiology but also pave the way for more effective treatments, ultimately benefiting the millions of individuals suffering from this prevalent and debilitating disorder.

## Abbreviations

CGRP: calcitonin gene-related peptide
CNS: central nervous system
ECoG: electrocorticography
FHM: familial hemiplegic migraine
KI: knock-in
mAbs: monoclonal antibodies
MGS: mouse grimace scale
MRI: magnetic resonance imaging
NMDA: N-methyl-D-aspartate
PACAP: pituitary adenylate cyclase-activating polypeptide
SD: spreading depolarization
